# Novel treatment of revision malarplasty with piezosurgery: A case report

**DOI:** 10.1097/MD.0000000000022529

**Published:** 2020-10-09

**Authors:** Qiang Zhang, Caiwang Chang, Zhibing Meng, Jinhua Huang, Jun Guo, Zili Ge

**Affiliations:** a Department of Maxillofacial Surgery, the Affiliated Hospital of Yangzhou University, Yangzhou University, Yangzhou; b Department of Oral and Maxillofacial Surgery, the First Affiliated Hospital of Soochow University, Soochow University, Jiangsu, P.R. China.

**Keywords:** osteotomy, piezosurgery, revisional malarplasty

## Abstract

**Background:**

Reduction malarplasty is a routine clinical procedure among Asian women, but the traditional surgical methods are still associated with serious complications, such as nonunion of the osteotomy sites. Revisional surgery to correct such complications is common, but poor bone healing in the osteotomy area presents a challenge to plastic surgeons. In this report, the authors present a new technique for revision malarplasty that uses the piezosurgery (piezoelectric bone surgery) approach.

**Patient and diagnosis:**

A 30-year-old female patient underwent reduction malarplasty with titanium plate fixation in the zygomatic region at another hospital 4 years ago, but the root of the zygomatic arch was not fixed. The patient was diagnosed with bone nonunion, facial asymmetry, and soft tissue sagging on the right side of the face after malarplasty.

**Intervention:**

We used piezosurgery to truncate the displaced healed broken end of the zygomatic bone according to the original osteotomy line. Following this, the malar was re-fixed with micro-titanium mesh, and the zygomatic arch was fixed with a titanium plate.

**Outcome:**

The patient was followed up for 11 months after the revision procedure. Her facial appearance was satisfactory, and no complications were observed on computed tomography images.

**Lessons:**

This report presents a novel therapeutic option for surgical revision of failed malarplasty. Piezosurgery can help overcome the limitations of traditional surgical methods by reducing bone resorption, preventing resorption of the bone in revision malarplasty, modifying the degree of inward and upward movement of the zygomatic bone by facilitating adjustment of the position of the drill hole in the cortex of the bone stump for stable fixation. Hence piezosurgery can be a simple, accurate, and non-invasive osteotomy method for revision malarplasty.

## 1 Introduction

One of the most recognizable features of the middle part of the face is the outline and protrusions of the upper lateral part of the cheek; it is referred to as the zygomatic eminence and is formed below the cheekbone.^[1]^ Prominent zygomatic body and arch is a common facial feature of Asian women that lends a wider appearance to their faces, but this is not considered to be in line with the mainstream aesthetics of oriental nationalities. As a result, reduction malarplasty, which is a surgical method for facial contouring, to change the outline of the middle face is one of the most common plastic surgeries among Asian women.^[2]^

With the increasing popularity and vigorous advances in malar reduction surgery in Asian countries, the number of cases requiring revisional malarplasty after zygomatic surgery has also been on the increase.^[3–5]^ The most common reasons for revisional malarplasty are facial soft tissue loosening, bone nonunion, and asymmetry, which have incidence rates of 2.8%, 2.2%, and 1.8%, respectively.^[3,4,6,7]^ In particular, bone nonunion can lead to zygomatic block displacement, damage to facial expression, facial droop, and depression. Nonunion can eventually lead to an increase in the size of the bone suture as a result of gradual absorption of the free broken ends on both osteotomy sides (due to the absence of stress stimulation), and this can make revision procedures more difficult.^[3,8]^ However, revision surgery after malarplasty is limited by the narrow intraoperative visual field and scar adhesion in the zygomatic bone area, which make it difficult to accurately control electric osteotomy tools and increase the risk of injury to blood vessels, nerves, soft tissue. In addition, deviation of the osteotomy line is highly likely, and repeated osteotomy could cause thermal damage to the bone and increase bone resorption, thus resulting in failure of the bone defect repair.^[4]^ In the past, bone nonunion was repaired via the coronal approach, but the risk of scar formation and hair loss is high with this method. Additionally, plastic surgeons typically use a chisel to cut the bone directly according to the original osteotomy line in order to avoid bone resorption caused by drilling near the crack, but this technique is associated with a high risk of fractures and bone fragmentation.^[3,4]^ Thus, the conventional methods for revision surgery have several drawbacks and risks.

**Figure 1:**
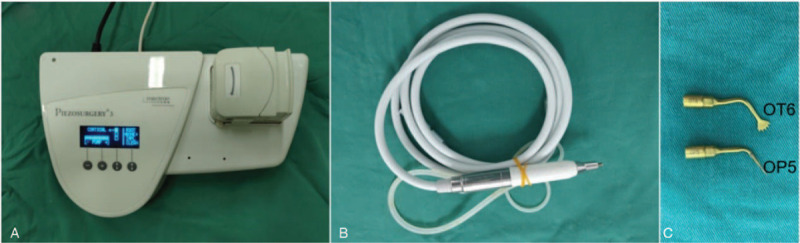
The Mectron piezosurgery system. (A) Main unit, (B) handpiece, and (C) ultrasonic device tips OP5 and OT6 (OT6 is designed for osteotomy, and OP5 is used to create a fixed point for insertion of the titanium nail).

As one of the recent advances in bone surgery, piezoelectric bone surgery (piezosurgery) has won the favor of surgeons because of its unique advantages. In this osteotomy method, high-frequency vibrations emitted from the tips of an ultrasonic device is used to generate a cutting force.^[9]^ Because the working frequency and tip amplitude are controlled within a certain range of 2-dimensional ultrasonic oscillation, damage to the surrounding soft tissue can be avoided via its absorption of a small amount of energy through elastic vibration. piezosurgery not only offers higher precision, but also reduces bone resorption and prevents re-resorption of the bone in revisional malarplasty; thus, it provides better accuracy and safety than traditional surgeries^[10,11]^ (Fig. 1). piezosurgery is used in a variety of applications such as sinus floor elevation, bone harvesting, and bone splitting, but its application in revisional malarplasty has not been reported to date.

Currently, there are many case reports on revisional malarplasty, but there are few reports on the techniques used for repair surgery. This paper is the first to describe a case where piezosurgery was used to repair bone nonunion after reduction malarplasty, and to describe the surgical technique that was used.

## 2 Case report

**Figure 2:**
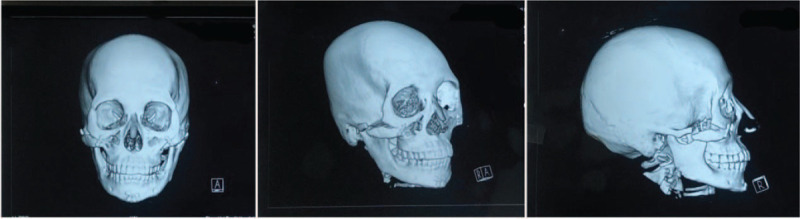
Preoperative computed tomography images of the patient showing bone malunion. The fractured root of the zygomatic arch was not fixed, and this resulted in downward movement of the bone mass and malunion of the bone mass in the right malar region.

**Figure 3:**
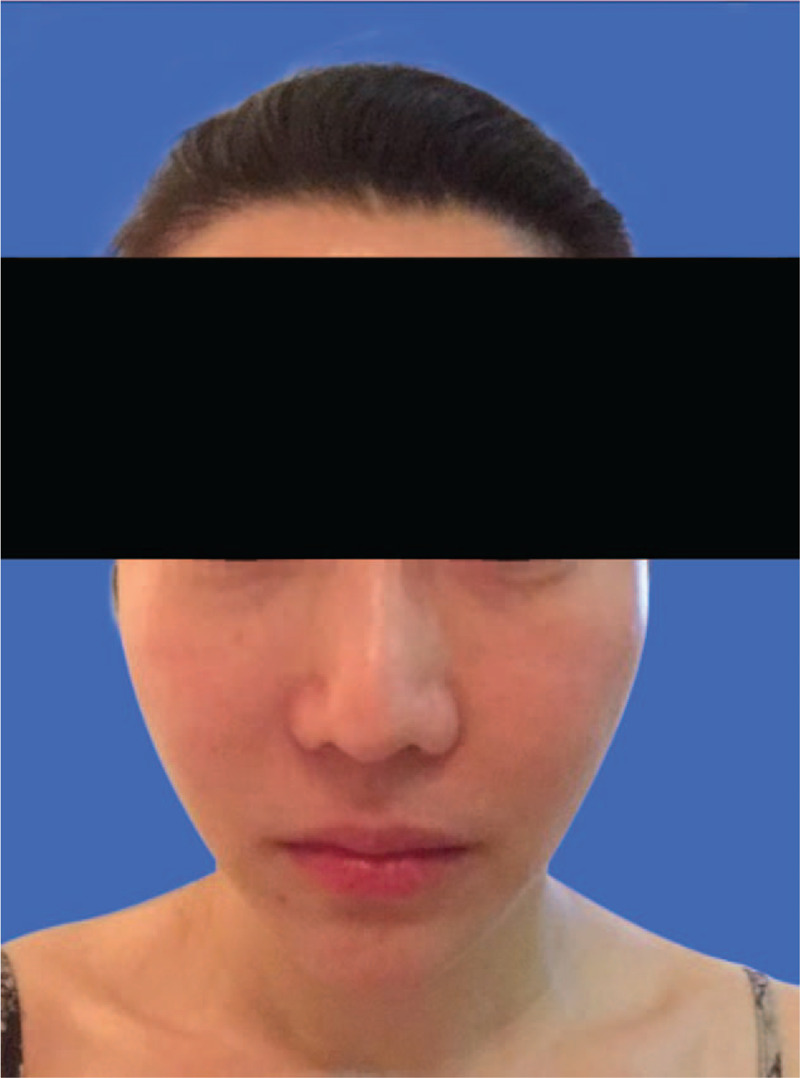
Preoperative photograph of the patient's face showing asymmetry. Due to downward movement of the bone mass, the muscle fascia attached to its surface moves downward together with it. This resulted in the loosening of the right facial soft tissue and facial asymmetry.

A 30-year-old patient presented at our hospital with bone malunion following reduction malarplasty that had been performed at another hospital 4 years ago. Macroscopic examination revealed that the root of the zygomatic arch was not fixed, and as a result, the bone mass had rotated downward due to the leverage force and shear force caused by the traction of the masseter muscle. As a result of the downward shift of the bone mass, the fractured end of the malar osteotomy section had been displaced and was healing poorly. Additionally, the space around the upper end was large, and a V-shaped bone defect was formed locally (Fig. 2). Moreover, as the bone mass moved downward, the muscle fascia attached to its surface moved downward along with it, resulting in the loosening of the right facial soft tissue and facial asymmetry (Fig. 3).

The patient consulted our hospital with the intention of having the facial internal fixation removed and the right zygomatic bone repaired. Before the procedure, we performed routine high-resolution multi-slice spiral computed tomography (CT) 3-dimensional imaging (slice thickness, 0.6 mm; interlayer spacing, 0.6 mm) to determine the position of the original osteotomy line and assess the extent of bone healing and bone deficiency, and accordingly, create a personalized bone repair plan.^[12]^ In the procedure that had been performed 4 years ago, the patient underwent reduction malarplasty with titanium plate fixation in the zygomatic region, along with chin osteotomy with titanium mesh fixation. According to the CT images, the chin and the osteotomy area of the left zygomatic bone had healed well. Additionally, the titanium plate and titanium mesh were not broken, so they could be used for secondary fixation. However, at the request of the patient, the original internal fixation material present in the chin and the left malar were removed, and revisional surgery of the right malar area was performed.

Procedure for right revisional malarplasty: The procedure was performed under general anesthesia and nasal intubation. We opened the intraoral tissue along the original incision and removed the internal fixation material. Following this, a 1-cm incision made in front of the auricle was used to fix the original osteotomy end of the zygomatic arch. The periosteal attachment on the surface of the original osteotomy area was removed, and the OT6 working tip of the piezosurgery device (Mectron piezosurgery; Mectron, Carasco, Italy) was used to separate the bone upward along the original osteotomy line up to the upper bone margin. The piezosurgery device was then used to truncate the zygomatic bone along the osteotomy line, and we moved up and pushed the bone block upward and inward to an appropriate position. The working tip OP5 of the piezosurgery device was then used to drill a point for insertion of a titanium nail. The internal fixation titanium MeSH that was removed from the chin was fixed on both sides of the osteotomy end of the zygomatic region. The periosteal attachment on the surface of the zygomatic arch of the original osteotomy region was removed; the zygomatic arch end was moved up to fold with the posterior osteotomy end; and the small titanium plate removed from the left zygomatic region was used to fix the anterior and posterior osteotomy ends. Following this, the zygomatic body and zygomatic arch were confirmed to be stable in the designed position, and extensive bony contact was observed between the osteotomy ends. Further, the presence of soft tissue between the osteotomy ends was ruled out. A 4-0 absorbable suture was used for closure of the intraoral incision, and a 6-0 aesthetic suture was used for closure of the anterior auricular incision. No arrangement for negative pressure drainage was made postoperatively. The operation lasted 1.5 hours and the bleeding volume was about 50 mL. We administered antibiotic treatment for 3 days and performed oral care. The patient was advised not to blow her nose as it might cause maxillary sinus hypertension and reflux of nasal contents to the surgical area.

**Figure 4:**
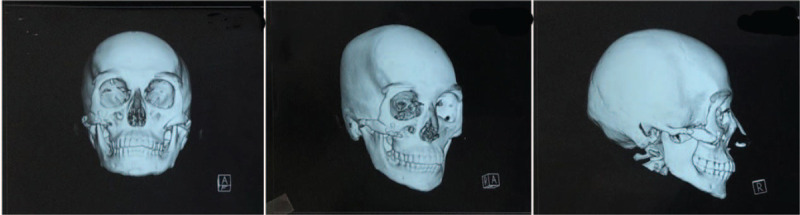
Computed tomography images of the patient showing repair of the bone defect at the 11-month review after revisional surgery. The internal fixations in the area of right zygomatic body and zygomatic arch were in place. Additionally, the bone mass was not displaced, and bone continuity in the operation area was corrected.

**Figure 5:**
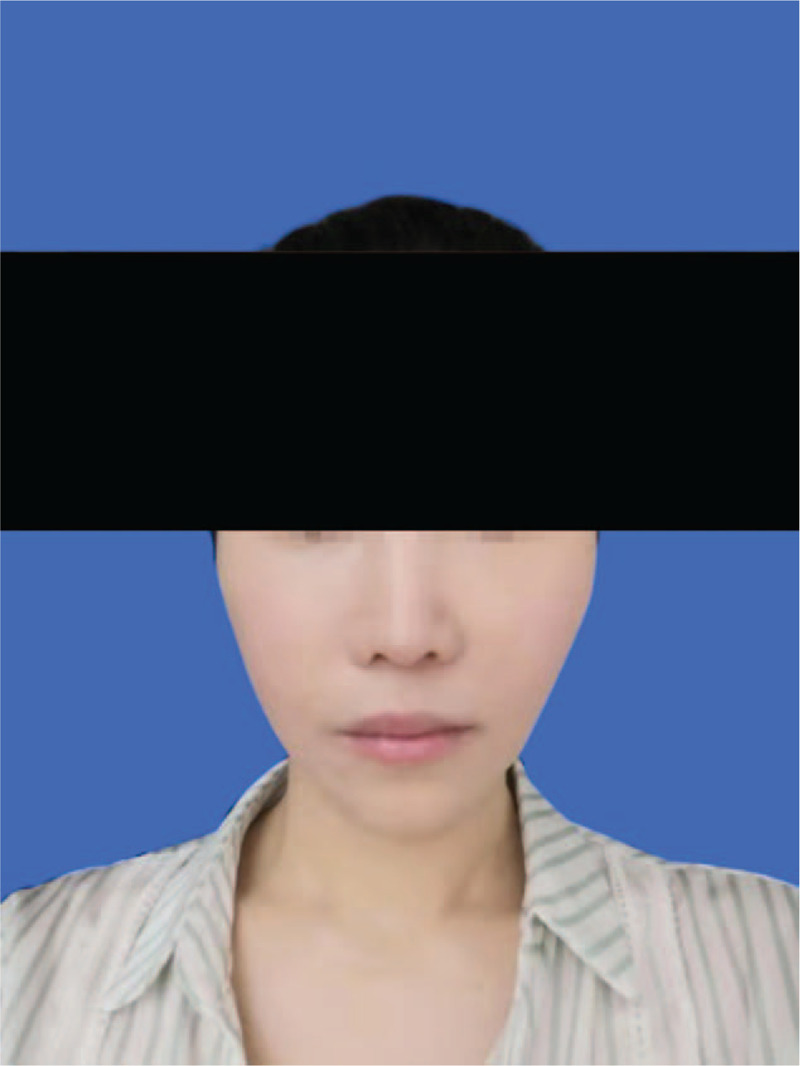
Photograph of the patient taken at the 11-month review showing correction of zygomatic collapse and facial asymmetry. The collapse of the right zygomatic face was significantly improved compared with that before the revisional surgery, and the soft tissue droop was corrected.

The procedure was considered to be successful, as the outcome was consistent with the preoperative design. Further, there were no complications during and after the surgery. At the 11-month follow-up review, 3-dimensional images were taken and compared with those taken before the surgery. The images showed that the internal fixations in the area of the right zygomatic bone and zygomatic arch were in place; further, the bone mass was reconnected as a whole and the mouth opening was normal (Fig. 4). Comparison of the preoperative and postoperative images also showed that the collapse of the right zygomatic face had significantly improved and the appearance of the curve was smooth. Thus, the overall outcome was satisfactory (Fig. 5).

## 3 Discussion

This report describes a case of bone malunion following malarplasty that was fixed with the help of piezosurgery. This is the first description of a revisional malarplasty technique with piezosurgery, so the findings are expected to be interesting and important to plastic surgeons.

Malunion of zygomatic bones after reduction malarplasty is a serious complication that is very difficult to correct.^[13]^ Currently, it is considered that the key to repair zygomatic nonunion is to remove the fibrous soft tissue present in the nonunion site, as this provides a fresh bone contact surface and releases the zygomatic complex. As a result, the osteotomy end of the bone can be fixed in the desired position. Qiu et al, using finite element analysis, found that malar pressure produces an obvious stress concentration region at the root of the zygomatic arch.^[14]^ Therefore, we believe that during the reconstruction of zygomatic bone, besides repairing the anterior fissure of the zygomatic bone, the posterior part of the zygomatic arch should also be fixed. Otherwise, the continuity of the zygomatic bones is poor after the revision, and the osteotomy end of the bone could be displaced and might rotate again due to movement of the masseter muscle in the later stage. In our case, the patient had facial asymmetry and nonunion: The zygomatic body and zygomatic arch root were amputated along the original osteotomy line, and the bone mass was pushed inward and reset upward. The zygomatic body was then fixed with a titanium mesh, while the root of the zygomatic arch was pushed inward and upward as far as possible so that it overlapped with and was fixed with the rear. In patients with large bone defects and nonunion after zygomatic reduction, autologous bone transplantation to repair the severed zygomatic body can be considered for restoring the continuity and physiological function of the zygomatic complex. Further, if the patient has severe facial asymmetry or relaxation, zygomatic facial soft tissue lifting for soft tissue repair could be considered.^[8,15]^

The approach to revisional malarplasty should be based on the unique features of each case, and an individualized plan should be created for each patient. For the treatment of bone resorption and poor bone healing, the first step to promote bone stump healing is to ensure stable fixation, and the prerequisite for stable fixation is sufficient bone contact. Based on our experience with the present case, the bone mass of the zygomatic area can be evaluated by CT before the procedure. If the CT findings indicate that the repair operation can prevent further bone loss, bone transplantation is not required.

The principle of piezosurgery is based on the difference in the acoustic impedance of bone tissue and soft tissue, as it is high in bone tissue and low in soft tissue. The cutting power in piezosurgery is derived from micro-oscillations, and the working frequency of an ultrasonic osteotome is 24 to 36 kHz. The swing amplitude of the tip is 60 to 200 μm in the horizontal direction and 20 to 60 μm in the vertical direction. There is a slight amplitude vibration that is invisible to the naked eye only at the tip, and the procedure requires only a small amount of strength. This allows for better control of the device, and helps to achieve micron bone cutting.^[9,10]^ In addition, in the process of cutting with piezosurgery, the temperature of the bone osteotome is controlled below 38 °C by washing with cooled saline. Therefore, the degree of thermal injury to the bone is kept at a minimum.^[16]^ Additionally, the postoperative healing time is shorter than that with other revision methods, and the pain and swelling are also alleviated. Another advantage of piezosurgery is the cooling and flushing system, which helps keep the field of vision clear and reduces bleeding in the operative area.^[16,17]^ To summarize, piezosurgery has the following advantages over the traditional bone cutting power system used in revisional malarplasty: low bone resorption, and prevention of thermal injury, irregular osteotomy, soft tissue injury and so on. As piezosurgery causes little damage, the biomechanical changes in the masseter muscle and zygomatic muscle are smaller and the complications related to the osteotomy are lower.^[5]^

In the present case, we used an OT6 working tip of the piezosurgery device for osteotomy. As piezosurgery offers selective bone cutting, the mucosa of the maxillary sinus could be peeled off with a stripper and dissociated from the bone surface to avoid damage. Thus, piezosurgery can effectively prevent maxillary sinus inflammation. In addition, the OP5 working tip of the piezosurgery device can modify the degree of inward and upward movement of the zygomatic bone by adjusting the position of the puncture in the cortex of the bone stump. Stable fixation can be achieved by adjusting the height of the perforation so as to avoid downward movement and valgus of the zygomatic bone.

To conclude, this paper describes in detail the design and procedure for revisional malarplasty with piezosurgery, and also discusses the prevention and correction of the complications of bone nonunion. Thus, this paper provides useful evidence-based information for plastic surgeons over the world.

## 4 Conclusion

Bone nonunion, facial asymmetry, and droop after reduction malarplasty are the main reasons for repair surgery. There are various approaches to revisional malarplasty, and in patients with malalignment and nonunion of zygomatic bones, the surgical approach should be designed according to the results of imaging examination. piezosurgery is ideal for osteotomy and positioning the perforation in revisional malarplasty, as it has better accuracy and safety, and is minimally invasive and offers a clear surgical field compared with traditional osteotomy methods. Additionally, it reduces the operating time and costs while providing optimal results.

## 5 Disclosure

The authors declared that there was no conflict of interest in the financial and publication of the work. All the authors gave their seal of approval to the manuscript.

## 6 Compliance with ethical standards

(1) The authors declare that they have no conflicts of interest to disclose.
(2) All procedures performed in studies involving human participants were in accordance with the ethical standards of the institutional and/or national research committee and with the 1964 Helsinki declaration and its later amendments or comparable ethical standards.
(3) This study is approved by the Medical Ethics Committee of the Affiliated Hospital of Yangzhou University.
(4) The patient in the case had given her informed consent in writing prior to inclusion in the study.

## 7 Consent for publication

Written informed consent was obtained from the patient for publication of this case report and accompanying images.

## Author contributions

**Formal analysis:** Zhibing Meng, Jun Guo.

**Investigation:** Jinhua Huang, Zhibing Meng, Jun Guo.

**Methodology:** Jun Guo.

**Resources:** Jun Guo.

**Writing – original draft:** Qiang Zhang, Caiwang Chang.

**Writing – review & editing:** Caiwang Chang, Jun Guo, Zili Ge.

## Abbreviations

Abbreviations: CT = computed tomography, piezosurgery = piezoelectric bone surgery.
